# Parent–adolescent communication on adolescent sexual and reproductive health in sub-Saharan Africa: a qualitative review and thematic synthesis

**DOI:** 10.1186/s12978-021-01246-0

**Published:** 2021-10-10

**Authors:** Ijeoma Usonwu, Raheelah Ahmad, Katherine Curtis-Tyler

**Affiliations:** 1 Nursing, City University of London School of Health Sciences, London, UK; 2 Division of Health Services Research and Management, City University of London School of Health Sciences, London, UK

**Keywords:** Adolescent, Parent, Sexual health, Sexuality, Sex education, Sex, Reproductive health

## Abstract

**Background:**

Improving adolescent sexual and reproductive health continues to be a global public health need. Effective parent–adolescent communication on sexual health issues has been cited as a factor that could influence adolescents towards adopting safer sexual behaviour. The current review synthesises qualitative literature to understand the nature and relevance of parent–adolescent sexual and reproductive health communication and the barriers to effective communication in sub-Saharan Africa.

**Methods:**

We systematically searched and synthesised qualitative literature published between 1st January 1990 to December 2019 and searched from CINAHL, PsycINFO, MEDLINE, Global Health, EMBASE, PubMed, and Google Scholar. We assessed the methodological quality of included studies using the Critical Appraisal Skills Programme (CASP) checklist. We thematically analysed qualitative data from the included primary studies.

**Results:**

Fifteen studies were included. Social and physiological events act as triggers for initiating discussions. Fear of personal, social, and economic consequences of high-risk sexual behaviours act as drivers for communication but also carry a negative framing which hinders open discussion. Lack of parental self-efficacy and cultural and religious norms create an uncomfortable environment leaving peers, media, teachers, and siblings as important and sometimes preferred sources of sexual health information.

**Conclusions:**

While mothers recognise their own role in adolescent sexual and reproductive health and school-based interventions can act as useful prompts for initiating discussion, fathers are mainly absent from home-based dialogue. Fear dominates the narrative, and the needs of adolescents remain unarticulated.

**Supplementary Information:**

The online version contains supplementary material available at 10.1186/s12978-021-01246-0.

## Plain English Summary

Improving adolescent sexual and reproductive health remains an important public health need globally. Effective communication on sexual health issues between adolescents and their parents has been recognised to influence safer sexual behaviour among adolescents. This review combined qualitative evidence to understand the nature of, and barriers to communication about sex between parents and adolescents in sub-Saharan Africa.

We searched academic databases for relevant articles published between January 1990 and December 2019 and found 15 studies of appropriate quality. Data on parents and adolescents’ experiences of sex communication in these studies was synthesised. Drivers for sex communication including fear of personal and economic consequences were highlighted. Results also identified barriers to communication such as cultural and religious norms and lack of parental knowledge and skills.

Findings showed that adolescents sometimes prefer other sources of sexual health information like peers, media, and siblings. Mothers are recognised to be more involved with home-based interactions on sexual health with adolescents compared to fathers. The results of this review point to the necessity for adolescents’ needs to be understood and articulated towards influencing policy and programmes.

**Supplementary Information:**

The online version contains supplementary material available at 10.1186/s12978-021-01246-0.

## Key messages

- Adolescents in sub-Saharan Africa remain at high risk for sexually transmitted infections, female genital mutilation, and early pregnancy.
- Adolescents mostly view sex-related discussions with parents as uncomfortable, and interactions are driven by fear of personal, social, and economic consequences of sexual risk-taking behaviour.
- Parents recognise their lack of capacity to engage with adolescents and are influenced by cultural norms and religious beliefs.
- Future research needs to address the current imbalance of adult versus adolescent voices in published work and also ensure a creation of space for conversations about sexuality with sexual and gender minority youth in the Lesbian, Gay, Bi-sexual, Transgender, Queer, Intersex, and Asexual communities.

**Supplementary Information:**

The online version contains supplementary material available at 10.1186/s12978-021-01246-0.

## Introduction

Maintaining and improving Adolescent Sexual and Reproductive Health (ASRH) continues to be of global public health importance, particularly as over a sixth of the world’s population are aged 10–19 years [1]. In sub-Saharan Africa (SSA), young people aged 10–24 years account for a third of the population [2]. An estimated 15 million adolescents get married before 18 years of age each year, with 90% of births within marriage recorded among 15 to 19-year-olds [3]. Adolescents living in SSA also bear the greatest burden of HIV/AIDS (89%) globally [4]. Other sexual and reproductive health (SRH) issues affecting adolescent girls in SSA, and which may contribute to high morbidity and mortality rates include unsafe abortions, complications during pregnancy and childbirth, and gender-based violence, including female genital mutilation [5]. High-risk sexual behaviours amongst adolescent boys in SSA lead to fatherhood during their adolescent years which can adversely affect mental health and wellbeing, occupational and educational opportunities [6].

Strategies to promote healthy adolescent sexual behaviour have ranged from influencing individual behaviours through sex education (school-based, peer education, community-based) and behavioural campaigns aimed to delay sexual debut and promote protective behaviours for when they are sexually active [7, 8]. Legislative measures include access to emergency and over-the-counter contraception and raising the age of consent for sex [9]. At a structural level, interventions aimed at addressing wider contextual factors include microfinance initiatives to empower adolescents economically [10]. There remains a need for comprehensive ASRH programmes which account for cultural and social influences including those from families, peers, and communities. There is also a need to explore how personal experiences and interactions with these immediate and wider environments shape attitudes and behaviours [11]. The conscious explicit and unconscious implicit communication, interactions, and observed norms within families can be powerful contributors in the socialisation of children and adolescents and also in regard to sexual behaviours [12]. While parents bear a responsibility of providing information and education to their children, monitoring their children’s activities, and providing support when required, parenting can be daunting; particularly during the physical, physiological, and emotional changes during puberty and adolescence [13].

Parents are in close proximity and regular contact with their children. Thus, they potentially have the opportunity to shape behaviours, provide guidance and influence understanding of risk [14, 15]. Evidence from the US context suggests that children of parents who adopt an authoritative and more hands-on parenting style are less likely to engage in risk behaviours [14, 16]. Additionally, that those that get sexual health information from parents or grandparents are more likely to delay sex [17]. Having a closer relationship with their children allows some parents to have open and honest exchanges about sexual health matters [15, 18]. Recommendations concur with this approach by targeting parents for enhanced communication skills for effective sexual health information exchange [18].

In SSA, previous research examining the role parents play in ASRH have enabled a better understanding of adolescents’ sexual socialisation [19]; determined the comparative effects of growing up in a household with a single parent or both parents on adolescent decision-making and sexual behaviour [20, 21]; and facilitated understanding of how parental characteristics including self-efficacy, education level, and parenting styles and presence influence adolescent sexual behaviour and overall sexual health [22].

The aim of this review was to provide an evidence synthesis on the nature and relevance of parent–adolescent sexual and reproductive health communication in SSA. The importance of adolescents’ voices in terms of study design and data collection, and in what is reported in the findings of studies was carefully examined with the aim of understanding the unique needs of adolescents [23]. This is important in informing appropriate intervention design and better uptake of such interventions. It is also vital for centrally guarding the rights of a child and adolescent to appropriate and accurate sexual health information and education [24]. Specifically, this review addressed three sub-questions: *What are parent and adolescent views, experiences, and preferences of sexual health communication? What are the facilitators and barriers to parent–adolescent sex communication? Which alternative sources of sexual health information are accessed by adolescents?*

## Methods

### Design

This qualitative review and thematic synthesis was guided by the Preferred Reporting for Systematic Reviews and Meta-Analyses extension for Scoping Reviews (PRISMA-ScR) [25], and Thomas and Harden’s thematic synthesis method [26]. This method of analysis included line by line coding, development of descriptive themes and the generation of analytic themes. This approach was suitable for exploring sexual health communication between parents and adolescents from multiple perspectives. It allowed identification of commonalities towards informing policy and for improved ASRH.

### Search methods

The PEO (Population, Exposure, Outcome) model was used to determine the key concepts in the topic, to define eligibility criteria, and define search terms. Search terms covered the population of interest; exposure (Parent and Adolescent Communication (PAC) on SRH; and context of this review (SSA) [27]. A systematic search of key health electronic databases available within two main hosts EBSCOhost (CINAHL, PsycINFO) and OVID Online (MEDLINE, Global Health, EMBASE, Soc. Policy and Practice) was conducted to find literature published in English. Other academic databases searched include African Journals Online, BioMedCentral, PubMed and Web of Science. Relevant search terms were utilised to broaden the search. Search terms included those relating to the population of interest “adolescent” such as “teen”, “teenager”, “juvenile”, and relating to “parent” such as “guardian”; as well as terms relating to the study outcome which in this review includes SRH-related terms such as “sexual health”, “sexuality”, “sex education”, “sex”, “reproductive health” as indicated in each database. Controlled vocabulary and free text terms for each electronic database were followed. Search terms were combined with a list of SSA countries as classified by the World Bank during the period of carrying out the search (Table 1). Grey literature was searched using Google Scholar, EtHOS, SCOPUS, and ERIC. Websites of relevant organisations, namely, WHO, UNICEF, UNCRC and Save the Children were also searched. Reference lists of eligible studies were hand-searched to identify any additional studies. The literature search was carried out between June and December 2019. The search was limited to articles published after 1st January 1990, the era of the Millennium Development Goals and Sustainable Development Goals and the following inclusion and exclusion criteria were applied (Table 2). The review protocol is available upon request.

**Table 1:** Search strategy-PsychINFO search

# SEARCH SYNTAX in PsycINFO	
Parent Terms	
S1: (MH "Parents") OR (MH "Single Parent") S2. AB parent* OR AB guardian S3. 1 or 2	
Adolescent terms	
S4. (MH "Adolescent") S5. AB adolescen* OR AB teenag* OR AB youth OR AB juvenile OR AB minor OR AB schoolgirl OR AB schoolboy S6. 4 or 5	
Sexual and Reproductive Health Terms	
S7. (MH "Sexual Health") OR (MH "Reproductive Health") OR (MH "Adolescent Health") OR (MH "Sexuality") OR (MH "Sexual Behavior") OR (MH "Unsafe Sex") OR (MH "Heterosexuality") OR (MH "Sexual Abstinence") OR (MH "Safe Sex") OR (MH "Sex Work") OR (MH "Puberty") OR (MH "Child Abuse, Sexual") S8. AB "sexual health" OR AB sex* OR AB sexuality OR AB "reproductive health" S9. 7 or 8 S10. (MH "Sexual Behavior") OR (MH "Unsafe Sex") OR (MH "Sexuality") OR (MH "Sexual Abstinence") OR (MH "Safe Sex") OR (MH "Sex Work") OR (MH "Adolescent Behavior") OR (MH "Child Behavior") OR (MH "Reproductive Behavior") OR (MH "Risk Reduction Behavior") OR (MH "Risk-Taking") S11. AB "sex* behavi?r" OR AB "risk* behavi?r" OR AB "sexual debut" S12. 10 or 11 S13. (MH "Sex Education") OR (MH "Sex Counseling") S14. AB "sex* education" OR AB "sex* health" OR AB "reproductive health" OR AB condom* OR AB contraceptive* OR AB "family planning" S15. 13 or 14	
Sub-Saharan Africa	
S16 Africa or Angola or Benin or Botswana or Burkina Faso or Burundi or Cameroun or Cape Verde or Central African Republic or Chad or Comoros or Congo Brazaville or Congo Democratic Republic or Cote d'Ivoire or Djibouti or Equitorial Guinea or Eritrea or Ethiopia or Gabon or The Gambia or Ghana or Guinea Bissau or Kenya or Lesotho or Liberia or Madagascar or Malawi or Mali or Mauritania or Mauritius or Mozambique or Namibia or Niger or Nigeria or Reunion or Rwanda or Sao Tome and Principe or Senegal or Seychelles or Sierra Leone or Somalia or South Africa or Sudan or Swaziland or Tanzania or Togo or Uganda or Western Sahara or Zambia or Zimbabwe	
S17 africa/ or "africa south of sahara"/ or central africa/ or east africa/ or sahel/ or southern africa/ or west africa/ or tropical africa/	
S18. 3 and 6 and 9 and 12 and 15 and 17

**Table 2:** Inclusion, exclusion criteria

Inclusion criteria	Exclusion criteria	Rationale
Population of Interest Adolescents (WHO definition: 10 to 19 years) Parents (biological, single or married, adoptive or custodial parents/legal guardians). Studies where with an age range wider than (10 -19), but the median age falls between 10 to 19 years	Young people outside the WHO definition. (Age: < 10 > 19) Where the median age is not between 10 to 19 years Non-legal guardians/caretakers	Aim of Study
Exposure (Area of Interest) PAC on ASRH; presents qualitative data from parents and/or adolescents regarding their communication on SRH matters	Sole focus on School-based sex education Sole focus on HIV/AIDS with no element of views and experiences of parents and adolescents regarding PAC on sex	Existing gap in evidence in SSA (No qualitative synthesis of evidence on PAC) Study aim
Outcome: Experiences and views on the nature, process of PAC on SRH	Smoking Drug or alcohol use	Study objectives
Study design: Primary qualitative studies employing but not limited to designs such as phenomenology, grounded theory, or ethnography Mixed studies with extractable qualitative element	Quantitative studies, Quantitative findings from mixed-methods studies, Randomised Controlled Trials without qualitative component	Aim of Study, Type of review
Study setting: Sub-Saharan Africa	North Africa (Algeria, Egypt, Libya, Morocco, Tunisia, Western Sahara, and South-Africa	Countries are not within Sub-Saharan Africa; South Africa is considered an upper-middle income economy by the World Bank (2014), therefore wider contexts influencing ASRH may differ from other countries in SSA
Language: Available in English Language	Not available in English Language	Availability and access
Time Period: 1st January 1990 utill date of the last database search	Pre 1st January, 1990	Published during the MDGs and SDGs period where clear goals for global maternal and child health were set
Type of Publication: Peer reviewed articles, Journal Articles, Research Reports, Theses or Dissertations	Policy Documents, Commentaries, and Opinion Papers	Study aim and objectives (Primary research finding required to answer questions)

### Study selection

Two reviewers (IU and KCT) screened title and abstracts and discussed any discrepancies. The literature screening process is represented in the PRISMA flow diagram [25].

### Quality appraisal

The CASP checklist for qualitative studies [28] was used to appraise the quality of the papers included in this review. First appraisal was carried out by one member of the review team (IU) and an agreement on the quality of papers was reached with one other member of the review team (KCT or RA). The CASP tool for qualitative studies consisting of ten questions was helpful for answering questions about the validity, results, and usefulness of each study. However, some studies could appear to be of good quality based on their methodological approach but falter in their process of data collection. Therefore, a scoring system was deemed unsuitable for appraising the papers in this study. Instead, the approach of this quality appraisal was to describe what had been observed using the CASP checklist as a guide, without excluding papers. This is because all studies were considered to have potentially valuable insights [29]. A weakness observed across half of the studies was non-reporting of dates of data collection. Although few studies only provided limited insight, all papers explored the questions to be answered in this review. All the fifteen papers included in this synthesis were published in peer reviewed journals and this was used as a marker for quality.

### Data extraction

Data extraction was carried out using a standard form in Excel (Additional file 1: Table S1) by the first reviewer (IU) and validated by the second reviewer (RA).

### Data analysis and synthesis

Thomas and Harden’s [26] methods for thematic synthesis were employed to uncover underlying meanings from qualitative data, develop analytical themes, and draw conclusions across studies. To achieve this, all articles were imported verbatim into NVivo12 for analysis.

An inductive line by line coding approach was adopted in this review after familiarisation with data in included studies [26]. This approach was preferred over extracting data based solely on review questions because some studies did not address the review questions directly. Therefore, the inductive approach to coding facilitated the capture of all data relevant to explore the review question. Coding was carried out in three phases: (i) Line-by-line coding of relevant qualitative data as free codes which were named according to the meaning and content and resulted in thirty codes; (ii) Free codes were examined to find relationships between codes and were subsequently grouped into six descriptive categories (iii) Based on the underlying meanings of descriptive categories, three analytical themes emerged for thematic synthesis.

Coding was conducted by reviewer IU, discussed with one other reviewer (KCT), and agreed by the review team (IU, KCT, RA). Analytical themes with their sub-themes were synthesised by assessing links and interconnectivities (Table 3).

**Table 3:** Analytical themes and meaning

Analytical theme	Meaning	Evidence from data
*Attributes of Sexual Health Communication* Sub themes: Content, Timing and Frequency, Comfort, Gender differences	Refers to characteristics of sexual health communication and relates to how parents and adolescents describe their experiences of communication sexual and reproductive health issues	“our parents started telling us about sex issues when we were about 12–14 years of age …Our mothers started telling us about these issues because they see us moving in the company of girls in the village… They tell us that our voices have started to be deep and we should not engage in sex with girls but to just be friends with them.”(Muhwezi et al., 2015).- Adolescent Example of Timing of sexual health communication (Nambambi and Mufune, 2011) “I am educated so while telling them about abstinence, unintended pregnancy, and STDs, I also discuss safe sex, boyfriends, condoms, and contraceptives with them. You may not believe it, but I tell them if they ever get pregnant, I should be the first to hear it. This way I do not lose much sleep over them”-Parent Example of Content (Izugbara, 2008)
*Facilitators and Barriers of Sexual Health Communication* Sub themes: Facilitators—importance, perceived benefits, impact on adolescent health Barriers—Parental factors (Parental absence, self-efficacy, socio-economic status, misconceptions), Trado-cultural norms and religious beliefs, Adolescent factors (Age, fear)	Describes underlying individual and wider level factors that facilitate and hinder sexual health communication between parents and adolescents as inferred from the data	“I do not want any of my daughters to stay in my house and get pregnant. A girl will not get pregnant if she does not have sex. That is why I discuss sex with them from time to time.”- Parent Example of perceived benefit as a facilitator (Izugbara, 2008) “The culture doesn’t allow us to talk to children about sex, especially the opposite sex”- Parent Example of Trado-Cultural norms as a barrier- (Kumi-Kyereme et al., 2014)
*Implications for adolescent sexual behaviour* Influence on adolescents’ decision making, Relevance of sex communication	Relates to the relevance of sexual health communication between parents and adolescents, and alternate sources of sexual health information that impact adolescents’ decision making regarding their sexual health	“Because there are so many diseases, and I will get pregnant…I fear it because I have nowhere to go if I get pregnant and also many people at home have high expectations in me” – Adolescent Example of influence on decision making (Wamoyi et al., 2010)

## Results

### Search results

**Fig. 1:**
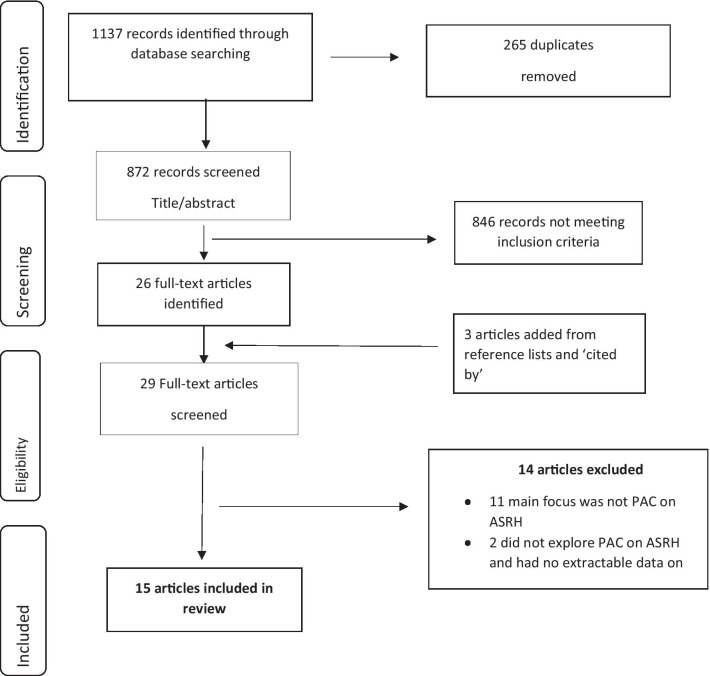
Prisma flow diagram

From the initial search 1137 records were returned, of which 265 were removed as duplicates. A further 846 were excluded based on title and abstract review. Three additional studies were identified from citation searches resulting in fifteen studies included in this study (Fig. 1).

The fifteen studies are fully described in Additional file 1: Table S1, including the study country setting, study design, sample size and characteristics, methods of data collection and analysis, and the main findings.

Studies were from Tanzania (5), Kenya (2), Ghana (2), Lesotho (2), and one from each of Nigeria, Zambia, Uganda, and Namibia. Data collection methods employed were focus group discussions (9), in-depth interviews (9), participant observation (3), and semi-structured interviews (3), with sample size ranging from 20 to 149. Four studies included parents, seven studies included both parents and adolescents (19 years median age), while two studies included only adolescent girls as participants. All studies used non-random sampling strategies including purposive sampling, snowballing sampling, criteria-based sampling, convenience sampling and fishbowl sampling. The analysis and synthesis of meanings, experiences, and preferences of SRH communication from these fifteen included studies resulted in two linked major themes: (i) attributes of SRH communication; (ii) drivers and barriers to SRH communication which had (iii) implications for adolescent sexual behaviour.

### Attributes of sexual and reproductive health communication

Attributes comprise of content, timing, and frequency of interactions between parents and adolescents, and their views on how comfortable these interactions feel. The relevance of gender is discussed within each sub-theme.

#### Content

This sub-theme includes the ‘what’ and ‘why’ of ASHR communication and conversations which were often broached in the context of morality, undesirable outcomes of sex, social consequences, and religious expectations. The narrative was centred around abstinence from sex until marriage, the negative direct consequences of engaging in pre-marital sex on adolescents’ health and indirect consequences on future social and economic prospects.

Adolescents felt the need for information and reassurance from parents about body changes during puberty and relationships. However, adolescents expressed that parents mostly resorted to negative tones including threats, demands, misinformation, warnings, and scare tactics about the dangers of sex to emphasise the need for abstinence [30–33]. Some of these discussions were consequent of parents’ religious (Christian) beliefs and cultural traditions which place expectations of chastity on adolescents [31, 32]. However, the reviewed literature did not include other religious beliefs such as Muslim beliefs. Adolescents expressed that they were dissatisfied with abstinence-only discussions [33]. Conversely, some adolescents reported more positive, open discussions about sex, communicated in friendly tones with counsel and advice [34].

Parents justified the primacy given in their conversations to health consequences of pre-marital sex such as HIV/AIDS and other sexually transmitted infections (STIs), unplanned pregnancy, and implications for adolescents’ educational and economic attainment and reputation in the community. Parents did not talk about sex as a natural experience or one to be enjoyed as this would undermine the case for abstinence [30, 32, 35–37]. Lack of other solutions or protective measures was further compounded by the lack of parental knowledge of STIs and the role of condoms and contraceptives [31, 38–40]. In some instances, parents expressed deliberately misinforming adolescents about condoms to create fear and discourage their use [34]. Other studies report open discussions on use of condoms and contraceptives were driven by the realisation that adolescents may get sexual health information from other sources and cannot be constantly monitored, as well as parents’ own experience of sexual exploration during their adolescence [36, 37, 41]. Conversely, parents’ own experiences of complete lack of SRH communication from their own adolescence was carried forward [34, 37].

#### Timing and frequency

**Fig. 2:**
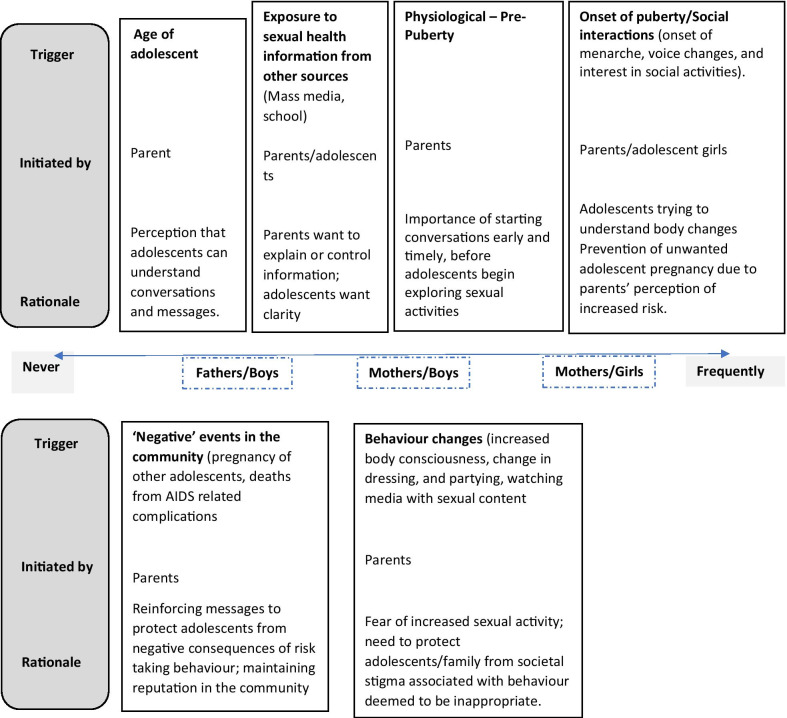
Rationale for the timing and frequency of parent–adolescent sex communication in the included studies

This sub-theme relates to the ‘when’, ‘how often’ and the ‘why’ of SRH communication which includes the prompts, as well as the importance of the timing of discussions. Figure 2 sets out the range from ‘never’ through to ‘frequently’, and the conditions for this. It also highlights the most commonly cited triggers which included social [31, 34, 40], physiological [34, 36, 37, 40], or community/school/media SRH interventions or campaigns [32, 34]. While parents acknowledged their responsibility for educating adolescents on SRH, and mothers in particular felt primarily responsible because of a closer relationship and understanding of needs [42], additional factors are relevant in influencing the timing and frequency of interactions (Fig. 2).

#### Comfort level

This sub-theme captures how comfortable adolescents and parents feel about SRH related communication. For adolescents this varied from being very comfortable, satisfied, excited, and hopeful, to being uncomfortable and bored. Overall, adolescents expressed a preference for conversations with their mothers compared to fathers [33, 34, 40], explained by the closer relationship with mothers. Their relationships with mothers who are considered main care givers, are described as ‘warm and open’ [33, 36, 40]. Both girls and boys overwhelmingly described sex communication with fathers as non-existent, rare, difficult, and uncomfortable, and distant relationships with fathers overall [36, 40, 43].

There was an overall feeling of comfort and trust in parents to give useful sexual health advise through conversations on topics they had life experience in, such as the onset of menarche. An important difference is reported between adolescents in school feeling able, inquisitive, and satisfied after engaging with their parents on sexual health problems [34], while those out of school feel unable to talk to their parents with feelings of embarrassment, and fear of parental judgement [30, 32, 36, 41]. This in turn exacerbated adolescent discomfort ranging from timidity, embarrassment, harshness, and caginess.

Parents reported difficulties arising from traditional constraints, lack of confidence in their ability to connect successfully with adolescents, lack of awareness of their child’s sexual knowledge and experience, fear of encouraging adolescents to begin sexual exploration, and lack of experience from their own parents to draw on [34, 36, 37, 40]. Parents even felt ‘ashamed’ talking about use of condoms and other contraceptives, particularly mothers with sons. Very much a minority, but some parents felt comfortable discussing sex with adolescents, because they felt strongly that they were the better source of sexual health information than mass media and peers [36]. However, parents still felt hindered because of the unease of adolescents [36, 37], thus requiring sensitivity, tact, warmth, and skill [30, 35].

### Drivers and barriers to sexual and reproductive health communication

#### Drivers for initiating ASRH communication were from perceived benefits, or conversely fear of consequences if conversations did not happen

Parents initiated discussions from a place of fear, and this also emanated into the content (as discussed above) [36, 37, 40, 41, 43]. As well as being a source of stigma to unmarried adolescents, unwanted pregnancy and abortion was seen as a source of shame to parents as they were often blamed for actions of their children. So, parents expressed that they were driven to initiate sexual health discussions because they were apprehensive of children bringing the family name to disrepute in the community [37, 42].

Perceived benefits and importance also motivated communication but this was largely from the perspective of adolescents. Receiving education from parents could help protect them from harmful sexual health related issues, and hence the need for open communication with parents [41].

Barriers were largely from parental lack of self-efficacy. This included the knowledge, language, and communications skills to address the sensitive topic of ASRH. For example, parents could not find the appropriate words in their local vernacular to describe anatomy or explicitly discuss sexual issues. Some rural parents explained that they had no idea how to approach sexual health discussions with their children [34, 37, 40]. Parental lack of confidence was fuelled by the perception that their children were more educated and already had more knowledge and experience with sexual health matters [34, 43]. Structurally, parental absence due to pressures of work and regular rural-to-urban travel for better economic prospects limited the opportunities for interaction [30, 34, 42, 44]. Negligence and de-prioritising family life were reported more for fathers compared to mothers [42]. Significant barriers to sexual health communication were associated with socio-economic status of households. Some parents with insufficient financial means to support their families felt highly compromised and did not question relationships of girls with (mostly older) men because of gifts and financial rewards that they received [43, 44].

Cultural and religious norms acted as drivers, but mainly as barriers to communication. Parents revealed that their culture and tradition did not allow parents to discuss sex with their children [30–32, 34, 35, 42]. Yet cultural norms also place expectations on parents to teach adolescents about sexual health issues when they come of age and facilitate discussions; but at the same time boys are largely excluded [37]. Modernisation, westernisation, and popularising of sexual issues were thought to encourage children to be sexually active, and hence the need for conversations with adolescents [37]. Urban and rural dwelling Christians affirmed that ASRH communication was against Bible teachings, and emphasised abstinence [31, 39]. Catholic parents were further conflicted in giving any contraceptive advice [42]. A minority did challenge tradition and norms to freely engage in communication about ASRH [42]. Norms such as economic benefits from daughter’s bride price and the desire to maintain family reputation also caused parents to monitor and control adolescents’ social associations [43]. The importance of guarding reputation sometimes acted as a driver for open discussions as well as hindering dialogue.

### Implications for adolescent sexual behaviour

#### Behaviours

The impact of parent–adolescent communication about sex results in two main outcomes. The first, to abstain and avoid relationships with the opposite sex. Adolescents explained that they had taken the decision to abstain from sex to meet high familial expectations and to please their parents [30, 40]. The second outcome which adolescents reported, was hiding sexual experiences from parents which might introduce a different set of challenges and defeats the aim of ASRH communication [30].

#### Sources of sexual health information

Besides their parents, adolescents also highlighted other relevant and preferred sources of sexual health information that also impact their sexual health decision making. Multiple studies in urban and rural settings identify school as an important source of sexual health information [30, 32, 33, 41, 42]. Adolescents felt that classrooms were a more relaxing, free, and open environment to learn about sexual health issues and expressed a preference for learning from school over mass media channels such as radio and the internet [32]. They further expressed that they acquired broader knowledge on sexual health issues from schools (teachers) compared to discussions with their parents because they had the opportunity to ask questions to help clarify confusion, read books, and share thoughts with peers. Adolescents also experienced getting more detailed information about teenage pregnancy and STIs such like HIV/AIDS, gonorrhoea, and syphilis from school HIV sensitisation campaigns and teacher-taught lessons [30, 32, 41, 42]. In addition, there were opportunities to learn associated skills such as how to use a condom from demonstrations in class [33]. Overall, adolescents reported positive experiences about sex education received in schools which enabled informed decisions about their sexual health.

Exchange of stories and experiences with peers, and advice for challenging situations were also valued, especially where parents did not broach the subject at all [30, 32, 33, 41]. In contrast to the home, adolescents expressed that at school they could talk about both positive and negative sexual health topics, particularly relationships, without reservations or fear of judgement [33]. Adolescents reported preferring to confide in their peers and teachers over their parents about relationships and other sexual health issues [32, 33]. While openness of communication with peers is valued, there was a recognition that peers may be an unreliable source of sexual health information [32]. Adolescents also identified health care centres and healthcare workers as sources of accurate sexual health information [33].

Adolescents preferred communicating with other relatives (i.e., grandmothers, uncles, and aunts) over their parents with comfort and protection from parental judgement [34, 41]. On matters concerning sexual or intimate relationships, adolescent girls expressed preference for talking to their sisters [41]. From parents’ observations, younger children preferred to get sexual health advice from their older siblings [37]. From a cultural context, traditional initiation ceremonies provided opportunities for sexual health information and teaching adolescents sexual norms and expectations in preparation for marriage [41]. However, these were mainly focused on female adolescents, emphasising gender stereotypes. Furthermore, adolescents felt that instructors at traditional ceremonies lacked adequate training, so they did not trust information received from them [32].

Adolescents living in urban areas shared experiences of engaging with sexual health programmes and adverts on television or radio which also served as prompts for parents to initiate discussions with them [32, 34].

## Discussion

**Fig. 3:**
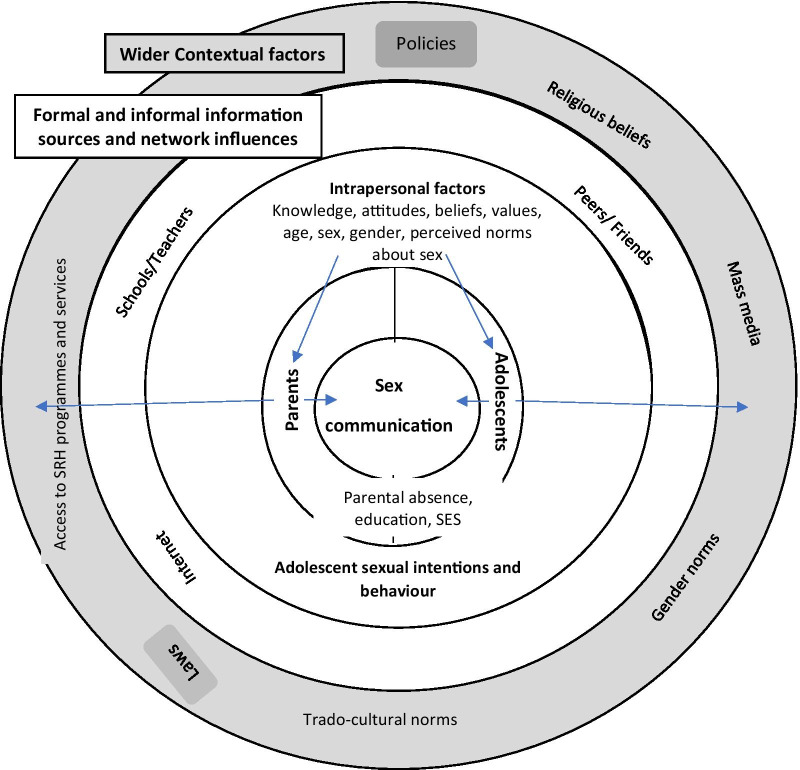
Conceptual framework for PAC

The findings support and build on a previous review on parent–adolescent communication in SSA [11], but which was dominated by quantitative studies. Our work provides a synthesis of new qualitative data and also explores alternative and preferred sources of sexual health information and their relevance for influencing adolescents’ decision making regarding their sexual and reproductive health. We present a conceptual framework for understanding the multi-level factors that impact parent–adolescent sex communication (Fig. 3).

Externally, laws and policies may influence availability and access to SRH information and services. Wider cultural and traditional factors along with formal and informal networks influence if, and how often parental and adolescent communication occurs. For example, the economic benefits from ‘daughter’s bride price’ and the desire to maintain family reputation encouraged them to monitor and control adolescents’ social associations. Additionally, at the household level, socio-economic status and levels of education have an impact. Counter to traditions, some low-income households would overlook relationships with their daughters which bought economic relief in the form of gifts. At the individual level, knowledge, and capacity to engage in conversations are variable and interconnected with parents’ own past experiences as adolescents. Evidence indicates that parents recognise this gap and are willing to undergo training to build their communication skills [30, 37].

The content of messages, timing and frequency of communication, parental and adolescents’ feelings about sex communication (comfort) and gender differences are reported. However, studies did not provide much insight into non-verbal forms of communication such as parental monitoring and connectedness. Content of exchanges were mainly focused on abstinence from sex, consequences of unsafe sex practices such as unplanned pregnancy, and sexually transmitted infections such as HIV/AIDS, and puberty-related changes. Evidence from other studies on sex education initiatives from both high and low resource settings demonstrate that receiving abstinence-only messages may predispose adolescents to more negative consequences of sexual risk behaviour such as unplanned pregnancy compared to receiving comprehensive sex education [45, 46]. Abstinence-only messages also violate adolescents’ fundamental rights to comprehensive and accurate health information [45]. Sexuality and sexual orientations were absent from conversations, in a context where national laws in several African countries criminalise individuals that identify in the LGBTQIA (lesbian, gay, bisexual, transgender, queer, intersex, asexual) community [47]. This also points to a violation of human rights and calls for the need for continuous advocacy towards decriminalising the LGBTQIA community. Information on STIs (other than HIV) such as gonorrhoea and syphilis was missing. When the risk of HIV/AIDS was discussed, testing and treatment were not. Information which acknowledged sexual activity such as adolescent-friendly sexual and reproductive health services and use of contraceptives/condoms was also avoided. While much of this is explained by cultural norms, a lack of education of parents themselves is a contributing factor [48]. But thinking outside of the context of SSA, adolescents in any setting may not approach their parents to talk about STIs and related issues because parents are unlikely to be experts in this field. Health experts have expressed that while it could be awkward, parents should engage their adolescents in talks about STIs as they could motivate safer sex choices [49, 50].

Late or ineffective communication, particularly after adolescents have begun sexual exploration, is unlikely to influence decisions to abstain from sex or practice safe sex. Recommendations from other studies indicate that sexual health communication may be more impactful among younger adolescents who are not fully sexually matured [7, 51, 52]. Parents preferred waiting so that messages could be understood [53–55]. As adolescents approach puberty, parents are prompted to share messages about physical and sexual maturation and expectations, but largely as a “one-off” conversation [55]. However, the importance of sustained interactions for better impact on adolescent’s behaviours is well known [56].

Gender, gender norms and gender roles determine the communicator and content of sexual health messages relayed to boys and girls, thus reinforcing gender stereotypes. Evidence from other cultures shows that sexual health communication between mothers and daughters impact sexual behaviour greater than father-son centred communication [15].

As in other cultural settings, religious beliefs (Christian) did hinder sexual health discussions as in Latino communities in the USA [57]. These indicate that adolescents may be missing out on vital information on safe sex practices which could be beneficial to their SRH as well as their overall health.

On the perceived influence of parent–adolescent sex communication on adolescent sexual behaviour, adolescents sometimes associated their decision to adopt safe sex behaviours with discussions that they had with parents or the parental monitoring of their social activities. These impacts are inconsistent across studies ranging from reduced sexual activity [58, 59], to greater [60]. This highlights the need for better consideration of how adolescents relate with their parents, closeness, and wider contextual factors such as cultural norms, religious beliefs, and values.

This review uncovered numerous barriers to parent–adolescent sex communication in SSA. Adolescents are exposed to information and misinformation from various sources. Some of these sources were highly beneficial because of the ease of access, levels of comfort, openness, and scope of information, particularly from schools (teachers), peers, siblings, and some mass media. On the other hand, some of these sources available via the internet such as social media can be harmful. Research is needed to explore the changing ways in which adolescents learn about sexual health issues, the major impacts on their decision making and influences of social media and the internet. They are faced with the challenge of deciding which information is accurate, negotiating expectations of parents versus those from their peers and making decisions about their sexual health with the information they receive. These alternative sources of information impose significant influence on adolescents’ beliefs, attitudes, values, expectations, behaviour, and decisions. Since mobile phones and internet sources are widely available in SSA and used, they may be a useful mechanism for interventions for parents and for adolescents to enhance knowledge, deliver training and a means of communication. Facilitators and barriers are mostly those expressed by parents and there is a critical need for more adolescent voices so that interventions are relevant.

### Strengths and limitations

Limitations of this work arise from consideration of English-only studies; other language studies may contain the gaps highlighted in this review such as fathers’ perceptions. The focus on qualitative studies only is a strength in terms of providing conceptual depth, but the findings are not generalisable beyond SSA. None of the studies identified included representation from respondents of other religious beliefs, for example Muslims, who account for about 30% of the population in SSA.

## Conclusion

This review has enabled further understanding of economic, cultural, and social influences on adolescent-parent interactions and communication about sexual health issues. The role of alternate sources of information and how these may affect their sexual health decision-making and behaviour is also considered. The review highlights parental recognition of their own potential role in communication about ASRH but also their lack of capacity to do so, especially fathers. The findings will benefit intervention design for adolescents, parents, and wider stakeholders. Our review has highlighted that the issues relating sexuality and sexual orientations are absent from conversations, and particularly pertaining to LGBTQIA. Future primary research should include experiences of sexual and gender minority adolescents (LGBTQIA) in the SSA region. With the theoretically informed conceptualisation of drivers and influences to adolescent and parental communication this study provides useful points of entry for future intervention design, evaluation, and research.

## Supplementary Information

**Additional file 1: Table S1.** Characteristics of Included Studies

## Data Availability

The list of publications used for the review are available in the manuscript and can be made available upon request.

## Abbreviations

ASRH: Adolescent sexual and reproductive health
CASP: Critical Appraisal Skills Programme
PAC: Parent-adolescent communication
PRISMA: Preferred reporting for systematic reviews and meta-analysis
SRH: Sexual and reproductive health
SSA: Sub-Saharan Africa
UNCRC: United Nations Convention on the Rights of the Child
UNICEF: United Nations Children’s Fund
WHO: World Health Organisation
