# Supplementary material for: Parent–adolescent communication on adolescent sexual and reproductive health in sub-Saharan Africa: a qualitative review and thematic synthesis
Source: Reprod Health. 2021 Oct 10;18:202. doi: 10.1186/s12978-021-01246-0 (PMC8504018; doi:10.1186/s12978-021-01246-0)
Supplement: Supplementary file 1 — Additional file 1: Table S1. Characteristics of Included Studies [file 12978_2021_1246_MOESM1_ESM.docx]

**Additional File 1 Table 3: Characteristics of Included Studies**

| **First author, year, County** | **Title** | **Study aim** | **Study design** | **Sample**  **N** | **Sample Characteristics** | **Sampling strategy** | | **Data collection method** | | | | **Analysis** | **Main findings** |
| --- | --- | --- | --- | --- | --- | --- | --- | --- | --- | --- | --- | --- | --- |
|  |  |  |  |  |  |  |  |  | FGD | IDI/KII | PO/SSI |  |  |
| Wanje, 2017  (Kenya) | Parents and teachers’ views on sexual health education and screening for sexually transmitted infections among in-school adolescent girls in Kenya: a qualitative study | To successfully develop and implement school-based sexual health interventions for adolescent girls | Qualitative | 26 | Parents  Age: 38-53yrs  80% female  Teachers  Age: 40-41yrs  100% female | Purposive sampling; schools | | ✓  4 | | ✓  10  IDI | __ | Content analysis | Parents have limited knowledge of STIs and seldom discuss them with adolescent girls. Teachers are more informed about STIs and together with parents, support STI screening for adolescent girls. Differences in parents or teachers’ gender, education level, religion and socioeconomic background did not impact their views about adolescent sexual health and acceptability of STI screening. |
| Wamoyi, 2010 (Tanzania) | Parent-child communication about sexual and reproductive health in rural Tanzania: Implications for young people's sexual health interventions | To explore parent-child communication about sexual and reproductive health.  Content, timing and reasons for their communication about sexual and reproductive health. | Ethnography | __ | Young people(YP)  Age: 14-24  Parents/caregivers  of YP aged 14-24 years. | Snowballing sampling (FGD); community  Purposive sampling (IDI); community | | ✓  17    YP  5 female  6 male  Parents  3 mothers  3 fathers | | ✓  46  IDI  (YP)  47% female  53% male  Parents  54% female  46% male | ✓  8  weeks | Thematic  Analysis  NVIVO 7 software | Content of sexual and reproductive health communication between parents and adolescents is mainly on abstinence, HIV?AIDS, unwanted pregnancy, marriage and focus on education. Parents initiate communication and focus more on female children. |
| Mturi, 2003  (Lesotho) | Parents' attitudes to adolescent sexual behaviour in Lesotho | To investigate the knowledge, attitudes and opinions of parents on various aspects of adolescents’ sexual and reproductive health | Qualitative | 222 | Parents of adolescents  50% Fathers  50% Mothers | Not explicit;  community | | ✓  30 | | __ | __ | Data analysis using NUD*IST (N4) software | Parents are often shy to discuss sex-related issues with adolescents. Some parents support sex education in schools while others think discussions about sex encourage adolescents to indulge in sexual activities. |
| Kajula, 2014  (Tanzania) | Dynamics of parent-adolescent communication on sexual health and HIV/AIDS in Tanzania | Qualitative exploration of sexual health communication with adolescents | Cross-sectional | 134 | 85% Adolescents  Age: 12-15  15% Parents | Criteria-based sampling | | ✓  12 | | ✓  20  IDI | __ | Analysis using interactive approach;  QSR Nvivo | Parents and adolescents agree that discussions about STI prevention methods such as condom use, pregnancy and family planning does not occur, although communication on some sexual health-related issues happen. Messages are usually based on fear. |
| Kumi-Kyereme, 2014  (Ghana) | Attitudes of gatekeepers towards adolescent sexual and reproductive health in Ghana | To discuss the views of adults on adolescent sexual and reproductive health | Qualitative | 60 | 40%Parents  Living with YP 12-19years/Community leaders  27%Teachers  33% Healthcare providers.  . | Not explicit.  Rural and urban communities: schools, healthcare institutions | | __ | | ✓  60  IDI | __ | QSR NG qualitative software | Parents acknowledged they were uncomfortable discussing sexual and reproductive health-related issues with their children. Community leaders feel adolescents are corrupted by sex education and provision of contraceptives by health workers and teachers. |
| Muhwezi, 2015  (Uganda) | Perceptions and experiences of adolescents, parents and school administrators regarding adolescent-parent communication on sexual and reproductive health issues in urban and rural Uganda | Describe the perceptions of adolescents, parents and school administrators about parent-adolescent communication on sexual issues | Qualitative | 149 | Adolescents/YP  Age: 12-20yrs  38% Male  42% Female  8% Fathers  5% Mothers  7% school administrators. | Purposive sampling;  schools | | ✓  11 | | ✓  10  KII | __ | Thematic analysis | Male and female adolescents communicate more frequently and openly with mothers than fathers. Fathers are stricter and often unapproachable or unavailable. Communication on SRH issues between parents and adolescents focused for on HIV/AIDS, STIs and body changes. Adolescents get SRH information from peers and mass media. |
| Nambambi, 2011  (Namibia) | What is talked about when parents discuss sex with children: family-based sex education in Windhoek, Namibia. | To explore parents’ communication with their children about sex | Qualitative | 36 | Young People  Age 14-25  56%  Parents/guardians  Of young people  44% | Purposive  sampling;  community | | __ | | __ | ✓  SSI  36 | Thematic analysis | Parent-children sex discussions are traditionally considered a taboo. Content of sex discussions include menstruation, HIV/AIDS and pregnancy. Both parents and adolescents agree that sex discussions are beneficial. |
| Wamoyi, 2011a  (Tanzania) | Socio-economic change and parent-child relationships: implications for parental control and HIV prevention among young people in rural North Western Tanzania. | To explore how socio-economic changes impact on parent-child relationships, particularly parental behavioural control and parental influence on YPs sexual behaviour. | Ethnography | __ | Young People  Age: 14-24yrs  Parents of YP aged 14-24 years. | Snowballing sampling and  Theoretical  Sampling  (FGD); community  Purposive sampling (IDI); community | | ✓  17  YP  5 female  6 male  Parents  3 mothers  3 fathers | | ✓  46  IDI | ✓  8  weeks | Anticipated and grounded codes. Thematic analysis.  QSR NVivo 7 software | Socio-economic changes such as education affect parent-child relationship dynamics, including their authority over YP’s sexual behaviour. Parents who depended on their children for material needs often gave ambiguous SRH messages to their children such as abstinence. |
| James, 2014  (Kenya) | Exploring the opinions of parents and teachers about young people receiving puberty and sex education in rural Kenya: a qualitative study. | To explore the opinions of teachers and parents in rural Kenya about delivering puberty and sex education and to identify their perceptions of barriers to young people accessing this education | Qualitative | 49 | Parents of children 9yrs or over  61%  Teachers  39% | Purposive sampling  Convenien-ce sampling;  schools | | __ | | __ | ✓  SSI  49 | Framework analysis | Several inconsistencies exist in the delivery of sex education in schools due to inconsistent methods that increase uncertainty among teachers. Parents believe that abstinence should be the main message of sex education programmes. Parent-child sex discussions center more around the dangers of pregnancy and HIV/AIDS rather than broader SRH information. |
| Krugu, 2016  (Ghana) | Who's that girl? A qualitative analysis of adolescent girls' views on factors associated with teenage pregnancies in Bolgatanga, Ghana. | To explore the psychosocial and environmental factors influencing the sexual decision making of adolescents | Qualitative | 20 | Adolescent girls  Age: 14-19yrs | Purposeful  homogeno-us sampling;  schools | | __ | | __ | ✓  SSI  20 | Thematic exposition using a  Grounded theory approach  NVivo 10  software | Adolescent girls talk about SRH issues with their mothers and friends. Much of mother-daughter communication was limited to moral advice to abstain from sex, or sometimes discussions on safer sex practices to avoid unwanted pregnancy. |
| Izugbara, 2008  (Nigeria) | Home-Based Sexuality Education: Nigerian Parents Discussing Sex with Their Children | To explore how and why parents in rural Nigeria discuss sexuality-related matters with their adolescent children | Qualitative | 73 | Parents  Fathers  Mothers of young people aged 10 and 21years. | Purposive sampling;  Fishbowl  Sampling;  Rural community | | __ | | ✓  IDIs  73 | __ | Thematic analysis | Very few rural parents discuss SRH issue with their children. Content od discussions focused on an unwritten code of cultural conduct to regulate YPs sexuality, sexual knowledge and their interest in sexual matters. |
| Wamoyi, 2011b.  (Tanzania) | Parental control and monitoring of young people's sexual behaviour in rural North-Western Tanzania: implications for sexual and reproductive health interventions. | To explore the dimension of parent-child control and monitoring and how this influences young people’s sexual behaviour. | Ethnography |  | Young People  Age: 14-24yrs  Parents/caregivers  of YP aged 14-24 years. | Snowballing sampling and  Theoretical  Sampling  (FGD); community  Purposive sampling (IDI); community | | ✓  17  YP  5 female  6 male  Parents  3 mothers  3 fathers | | ✓  46  IDI  (YP)  47% female  53% male  Parents  54% female  46% male | ✓  8  weeks | Combined approach to analysis using anticipated and grounded codes. Thematic analysis.  QSR NVivo 7 software | Parents are motivated to monitor and control children’s sexual behaviour for immediate and future economic and social benefits, like marriage. Patriarchal conditions that socialised male children different from girls contribute to gender differences in parental control. |
| Mturi, 2005  (Lesotho) | Perceptions of sex education for young people in Lesotho | To identify the views of young people, parents and teachers concerning sex education in Lesotho. | Qualitative |  | Adolescents  Age: 12-16  Parents: with at least one adolescent child  Teachers | Not mentioned | | ✓  46  Adolescents  10  Parents:  30  Teachers  6 | | __ | __ | Thematic analysis  NUD*IST  software | Parents discussions with adolescent, mostly girls, focused on avoidance of sexual behaviour using social consequences, religion or unwanted outcomes of sexual behaviour. |
| Butts, 2018  (Zambia) | HIV knowledge and risk among Zambian adolescent and younger adolescent girls: challenges and solutions | To identify sources of HIV prevention knowledge among young women aged 10- 14 years and community-based strategies to enhance HIV prevention in Zambia. | Qualitative | 114 | Adolescents  Girls 10-14yrs | Purposive sampling;  Schools,  community | | ✓ | | __ | __ | Thematic analysis  via  Grounded theory | Communication between parents and children on sex and SRH issues was deficient. Adolescents expressed discomfort in discussing sex with their parents and suggested that they were open to getting information sex and HIV/AIDS from parents. |
| Kajula, 2016  (Tanzania) | Parenting practices and styles associated with adolescent sexual health in Dar es Salaam, Tanzania. | To explore parenting practices and styles associated with adolescent sexual health in Tanzania | Qualitative | 24 | Adolescents  Age: 12-14yrs  Parents of adolescents | Criteria-based  Sampling;  schools | | __ | | ✓  IDI  24 | __ | Content analysis and comparative analysis  QSR NVivo | Most parents used an authoritarian style of parenting to control adolescents’ behaviour. Parents hope restrictions will help protect children from engaging in early sex. |
